# Dendritic Cells Require PINK1-Mediated Phosphorylation of BCKDE1α to Promote Fatty Acid Oxidation for Immune Function

**DOI:** 10.3389/fimmu.2019.02386

**Published:** 2019-10-15

**Authors:** Farhan Basit, I. Jolanda M. de Vries

**Affiliations:** ^1^Department of Tumor Immunology, Radboud University Medical Center, Radboud Institute for Molecular Life Sciences, Nijmegen, Netherlands; ^2^Department of Medical Oncology, Radboud University Medical Center, Nijmegen, Netherlands

**Keywords:** toll like 7/8 receptors, dendritic cell metabolism, branched chain amino acid (BCAA), PINK1, fatty acid oxidation (FAO)

## Abstract

Dendritic cell (DCs) activation by Toll-like receptor (TLR) agonist induces robust metabolic rewiring toward glycolysis. Recent findings in the field identified mechanistic details governing these metabolic adaptations. However, it is unknown whether a switch to glycolysis from oxidative phosphorylation (OXPHOS) is a general characteristic of DCs upon pathogen encounter. Here we show that engagement of different TLR triggers differential metabolic adaptations in DCs. We demonstrate that LPS-mediated TLR4 stimulation induces glycolysis in DCs. Conversely, activation of TLR7/8 with protamine-RNA complex, pRNA, leads to an increase in OXPHOS. Mechanistically, we found that pRNA stimulation phosphorylates BCKDE1α in a PINK1-dependent manner. pRNA stimulation increased branched-chain amino acid levels and increased fatty acid oxidation. Increased FAO and OXPHOS are required for DC activation. PINK1 deficient DCs switch to glycolysis to maintain ATP levels and viability. Moreover, pharmacological induction of PINK1 kinase activity primed immunosuppressive DC for immunostimulatory function. Our findings provide novel insight into differential metabolic adaptations and reveal the important role of branched-chain amino acid in regulating immune response in DC.

## Introduction

Dendritic cells (DCs) are specialized antigen presenting cells (APCs) in the immune system (1). DCs are central to pathogen sensing *via* an array of pathogen recognition receptors e.g., toll like receptors (TLRs) and stimulating antigen-specific T cells to proliferate and differentiate into effector and memory cells (2). Ligand binding to TLRs leads to DC activation and an enhanced capacity to stimulate T cells. DC activation is intrinsically linked to metabolic reprogramming (3). Currently, DC metabolism is mostly studied in the murine system, primarily using TLR4 agonist lipopolysaccharide (LPS) (4–7). Limited knowledge is available about the metabolic switch in human DCs (8–10). Furthermore, it is unclear whether all stimuli induce glycolysis in human DCs. In mice, TLR stimulation upregulates glycolysis, contributing to the metabolic requirements of high protein synthesis, a prerequisite for DC immune function. The TLR-stimulated “glycolytic burst” triggers *de novo* fatty acid synthesis through glucose-to-citrate metabolism, which is indispensable for DC function (4, 5).

Mitochondria are crucial for metabolic function. PTEN-induced putative kinase 1 (PINK1) predominantly localizes to the outer membrane of the mitochondria (11, 12) and is linked to mitochondrial function and subsequent metabolism (13). *PINK1* encodes a 581 amino acid protein with an N-terminal mitochondrial targeting sequence (MTS), a transmembrane domain (TMD), followed by a serine/threonine kinase domain. PINK1 is imported into mitochondria *via* the TOM or TIM23 complexes at the outer or inner mitochondrial membrane, respectively. Subsequently, its MTS is cleaved off by the mitochondrial processing peptidase located in the matrix. Afterwards, the inner mitochondrial membrane protease presenilin-associated rhomboid-like protease (PARL) cleaves PINK1 within the hydrophobic TMD between amino acids Ala103 and Phe104 (14–19). This generates 52 kDa N-terminally processed PINK1, which is released to the cytosol, where it is rapidly degraded by the proteasome through the N-end rule pathway (20). PINK1 is required for various cellular processes, e.g., regulation of mitochondrial bioenergetics through modulating complex I activity, promotion of mitophagy of depolarized mitochondria, protection against cell death, and protection of mitochondria via mitochondria-derived vesicles (MDV) (21).

Amino acids are the building blocks of proteins in mammals and their availability is of fundamental importance for cell survival, maintenance, and proliferation (22). Amino acids (23), especially glutamine (24–26), arginine (27, 28), and sulfur-containing amino acids (29) are of importance for the functioning of immune cells. Much less is known about the impact of branched-chain amino acids (BCAAs), valine, leucine, and isoleucine, on cells of the immune system. BCAA-transaminase converts BCAA into their corresponding branched-chain α-ketoacids (BCKAs). Then, BCKAs are catabolized by the branched-chain α-ketoacid dehydrogenase (BCKDH) complex within the mitochondrial matrix. Subsequently, acetyl-CoA is formed by metabolic reactions and incorporated into the tricarboxylic acid (TCA) cycle, or it enters the complex II of the electron transport chain (30). This process contributes to ATP production, which under physiological conditions is produced by OXPHOS. We here confirm previous results that a shift from OXPHOS to glycolysis occurs in human DCs stimulated with TLR4 agonist LPS (6). However, upon TLR7/8 stimulation of human DC we did not observe glycolysis. Instead we observed increased OXPHOS, which required FAO for immune function. These observations propose a scenario in which individual stimuli induce distinct metabolic reprogramming.

## Materials and Methods

### Chemicals

Antimycin A (#A8674), Oligomycin A (#O4876), Rotenone (#R8875), Kinetin (#48130), Etomoxir (#E1905), Lipopolysaccharides from *Escherichia coli* 0111:B4 (#L4391), and 3,6-Dichloro-benzo[b]thiophene-2-carboxylic acid (#ENA018104907) were obtained from Sigma-Aldrich. MitoTracker™ Green FM (#M7514), and 2-NBDG (#N13195) were obtained from Thermo Fisher Scientific.

### Human DC Culture and Stimulation

Human monocyte-derived DCs were differentiated from peripheral blood monocytes as follows. Buffy coats were obtained from healthy volunteers (Sanquin, Nijmegen, Netherlands) according to institutional guidelines. Peripheral blood mononuclear cells (PBMCs) were isolated by using Ficoll density centrifugation (Lymphoprep; Axis-Shield PoC AS, Oslo, Norway). Monocytes were isolated from peripheral blood mononuclear cells (PBMC) by adherence, as described previously (31) and cultured in complete DC medium supplemented with human recombinant GM-CSF (20 ng/mL) plus human recombinant IL-4 (20 ng/mL) (Peprotech) for 7 days. To generate immunosuppressive DC, 10^−6^ M Dexamethasone was added at day 3 in culture. On day 6 or 7, DCs were harvested, stimulated with LPS or protamine-RNA (pRNA) complex, which was made freshly before being added to the cells. Protamine (protaminehydrochloride MPH 5000 IE/ml; Meda Pharma BV Amstelveen, Netherlands) was diluted to 0.5 mg/ml in RNase free water and mixed with 2 kbp-long single-stranded mRNA (coding for gp100). It was extensively mixed and incubated for 5–10 min at room temperature, before adding to the cells.

### siRNA Transfection

For RNA interference, cells were transfected with SilencerSelect siRNA (Invitrogen), control siRNA (4390843), and siRNA targeting PINK1 (#s35166 and #s35168). At day 4 of the culture, the cells were harvested, washed with PBS, brought to a concentration of 1 × 10^6^ cells/100 μL resuspension buffer, and finally, transfected by electroporation with either 10 nM anti-PINK1 siRNA (#s35166) in combination with 10 nM anti-PINK1 siRNA (#s35168) or 20 nM control siRNA using the Neon transfection system (Invitrogen), according to the manufacturer's instructions. Electroporation was carried out with an electroporator (Neon with pipette station; Invitrogen) using three pulses (10 ms pulse width; 1350 V). After electroporation, cells were taken up in 10% FCS basal media without antibiotics and plated at 200 cells/μL. The next morning, the media was re-supplemented with penicillin, streptomycin, rGM-CSF, and rIL-4. At day 6, the cells were harvested, stimulated, and analyzed. Silencing efficiency was determined by western blot and qPCR of day 6 cells.

### Cytokine Detection

Supernatant was taken from each sample after incubation and analyzed with standard sandwich ELISAs to detect TNF-α using human TNF-α ELISA Kit (#88-7346-22) from Thermo Fisher Scientific.

### Flow Cytometry

Cell viability was determined using Fixable Viability Dye eFluor™ 780 (Invitrogen # 65-0865-14) according to manufacturer's instructions. Briefly, cells were incubated with Fixable Viability Dye eFluor™ 780 (1:2,000) at 4°C for 20 min. Subsequently, cells were washed and analyzed by flow cytometry. The following primary monoclonal antibodies (mAbs) were used to determine the maturation state of the DCs: anti–CD80- APC, anti–CD40-APC (all BD Bioscience, San Jose, CA). Measurements were performed on FACSVerse flowcytometers (BD).

### Metabolism Assays

An XF-96 Extracellular Flux Analyzer (Seahorse Bioscience) was used for Extracellular flux analyses of Dcss (50,000 cells/well) (32). For mitochondrial fitness tests, OCR was measured sequentially at basal, and following the addition of 1 μM oligomycin, 3 μM FCCP (fluorocarbonyl cyanide phenylhydrazone), 1 μM ROT+1 μM AA. Fatty acid oxidation was determined by monitoring the OCR of cells by using a FAO inhibitor, etomoxir (40 μM) in substrate limited medium. FAO was quantified as a response to etomoxir treatment as previously described (33, 34). For the glycolysis stress test, ECAR was measured sequentially at basal, and following the addition of 10 mM glucose, 1 μM oligolmycin and 50 mM 2-DG. Intracellular concentrations of Branched Chain Amino Acid (BCAA) were measured using colorimetric BCAA Assay Kit (#MET-5056, Cell Biolabs), as per manufacturer's instructions. Contribution of OXPHOS and glycolysis to ATP production was calculated as previously described (35).

### Quantitative Real-Time PCR (qPCR)

qPCR was carried out in a 25-μl reaction mixture containing 2 μl of cDNA, 12.5 μl of SYBR Green master mix (Applied Biosystems #A25742, Austin, USA), and 250 nmol of forward and reverse primer. The reaction conditions were as follows: 50°C for 2 min, 95°C for 10 min, and then 40 cycles of 95°C for 15 s and 60°C for 1 min. For qPCR, the primer sequences used are listed in Supplementary Table 1.

### Cellular ATP Measurements

The levels of ATP were assessed using ATP Bioluminecence Assay Kit CLS II (#11699695001, Roche GmbH, Mannheim, Germany), according to the manufacturer's protocol.

### Western Blot Antibodies and Detection

The PVDF membrane was probed with rabbit anti-PINK1 (#6946P; Cell Signaling), rabbit anti- Phospho (S293) BCKDE1A (#BET A304-672A-M; Bethyl Laboratories), anti-BCKDE1A (H-5) (#sc-271538; Santa Cruz Biotechnology) and mouse β-actin (#A5541; Sigma-Aldrich), followed by goat-anti-mouse or goat-anti-rabbit labeled with IRDye infrared dyes (LI-COR Biosciences, Leusden, Netherlands). A LI-COR Odyssey CLX infrared imaging system (LI-COR) was used for fluorescence detection.

### Statistical Analysis

Comparisons for two groups were calculated using unpaired 2-tailed Student *t*-tests with Microsoft Excel. A 2-way ANOVA with a Bonferroni post-test was used for comparison of more than two groups using GraphPad Prism 6 software (GraphPad). Differences were considered significant at *P* < 0.05.

## Results

### TLR Agonists Differentially Engage Metabolic Pathways in Human Dendritic Cells (DC)

**Figure 1:**
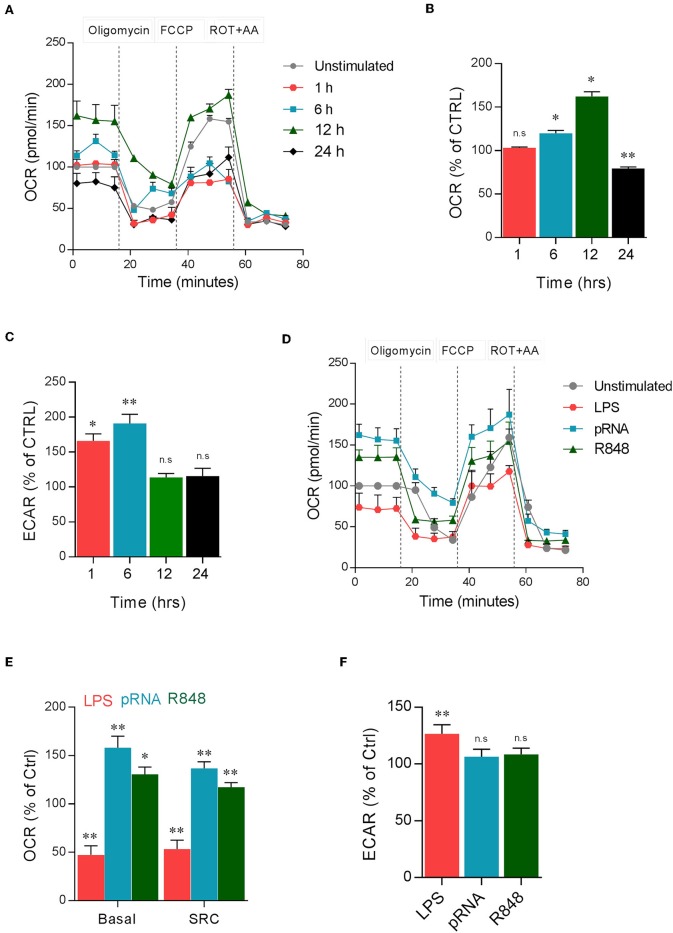
TLR ligands induced metabolic changes in DC. **(A)** Mitochondrial fitness test of DCs stimulated with pRNA for indicated time points. Data represents mean ± SEM of three independent experiments. **(B,C)** Data was collected within same experiments as **(A)**, but is shown separately for better understanding. Data represents mean ± SEM of three independent experiments performed in triplicate. ^*^p < 0.05; ^**^p < 0.01 (two-way ANOVA). **(D)** Mitochondrial fitness test of DCs stimulated with LPS (100 ng/mL), pRNA and R848 (1 μg/ml) for 12 h. Data represents mean ± SEM of three independent experiments performed in triplicate. **(E,F)** Data was collected within same experiments as **(D)**, but is shown separately for better understanding. Data represents mean ± SEM of three independent experiments performed in triplicate. ^*^p < 0.05; ^**^p < 0.01 (two-way ANOVA).

To study metabolic adaptations, human DC were stimulated with TLR7/8 ligand pRNA in a time-dependent manner. pRNA stimulation of DC increased oxygen consumption rate (OCR) after 1, 6, and 12 h of stimulation but decreased OCR after 24 h of stimulation (Figures 1A,B). Analysis of extracellular acidification rate (ECAR) revealed that pRNA stimulation increased glycolysis after 1 and 6 h but not after 12 and 24 h of stimulation (Figure 1C). Given the maximum increase in OCR and absence of glycolysis at 12 h after pRNA stimulation, 12 h time point was chosen for further experiments. Next, DCs were stimulated with three different TLR ligands for 12 h. LPS (TLR4), R848 (TLR8), and pRNA (TLR7/8). LPS stimulation of DCs decreased basal OCR, spare respiratory capacity SRC (Figures 1D,E), and ATP-linked respiration (Supplementary Figure 1A). This indicates induction of glycolysis which was underscored by the increase of ECAR (Figure 1F) upon LPS stimulation. In contrast, pRNA and R848 stimulation of DCs increased basal OCR, SRC (Figures 1D,E), and ATP-linked respiration indicating upregulation of OXPHOS (Supplementary Figure 1A). pRNA and R848 stimulation did not increase glycolysis, as ECAR did not change (Figure 1F). Furthermore, no increase in 2-NBDG uptake was observed in pRNA-stimulated DC (Supplementary Figure 1B) also demonstrating the absence of glycolysis induction.

**Figure 2:**
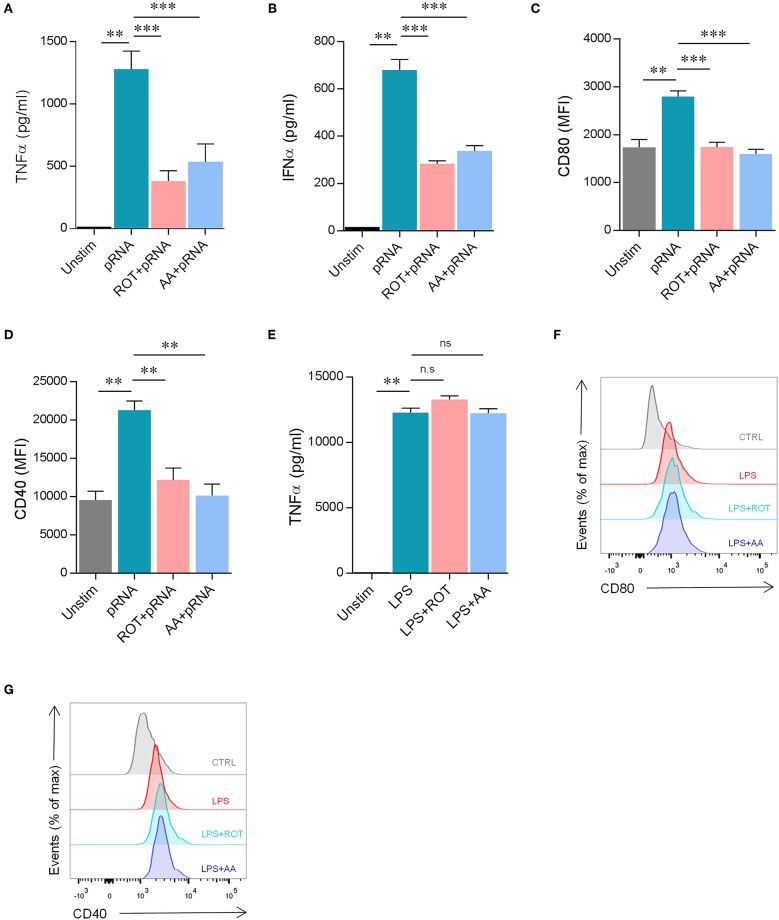
pRNA stimulation-induced OXPHOS is required for DC maturation. **(A)** TNFα levels on protein level were measured in the supernatant of the DCs stimulated with pRNA for 12 h in the presence or absence of 10 nM rotenone or 10 nM antimycin A. Data represents mean ± SEM of three independent experiments performed in triplicate. ^**^p < 0.01; ^***^p < 0.001 (Student's *t*-test). **(B)** IFNα levels on protein level were measured in the supernatant of the DCs stimulated for 12 h with pRNA in the presence or absence of 10 nM rotenone or 10 nM antimycin A. Data represents mean ± SEM of three independent experiments performed in triplicate. ^**^p < 0.01; ^***^p < 0.001 (Student's *t*-test). **(C)** Mean fluorescence intensity of CD80 in DCs stimulated for 12 h with pRNA in the presence or absence of 10 nM rotenone or 10 nM antimycin A. Data represents mean ± SEM of three independent experiments performed in triplicate. ^**^p < 0.01; ^***^p < 0.001 (Student's *t*-test). **(D)** Mean fluorescence intensity of CD40 in DCs stimulated for 12 h with pRNA in the presence or absence of 10 nM rotenone or 10 nM antimycin A. Data represents mean ± SEM of three independent experiments performed in triplicate. ^**^p < 0.01 (Student's *t*-test). **(E)** TNFα levels on protein level were measured in the supernatant of the DCs stimulated with LPS (100 ng/mL) for 12 h in the presence or absence of 10 nM rotenone or 10 nM antimycin A. Data represents mean ± SEM of three independent experiments performed in triplicate. ^**^p < 0.01 (Student's *t*-test) **(F)** Expression of CD80 on DCs stimulated with LPS (100 ng/mL) for 12 h in the presence or absence of 10 nM rotenone or 10 nM antimycin A. One experiment of 3 is shown. **(G)** Expression of CD40 on DCs stimulated with LPS (100 ng/mL) for 12 h in the presence or absence of 10 nM rotenone or 10 nM antimycin A. One experiment of 3 is shown.

The absence of glycolysis induction and the upregulation of OXPHOS in pRNA-stimulated DCs led to the question of whether OXPHOS is required for proper maturation of DCs. Upon activation, immature DCs undergo maturation which is crucial for effective antigen presentation and initiation of the primary immune response. Maturation of DCs is characterized by the high expression of antigen-presenting and costimulatory molecules and the production of pro-inflammatory cytokines. Here, the secretion of pro-inflammatory cytokine TNFα and IFNα and membrane expression of costimulatory molecules CD40 and CD80 was assessed. Inhibiting OXPHOS by Rotenone (ROT) or Antimycin A (AA) significantly reduced both the secretion of TNFα and IFNα (Figures 2A,B) and the upregulation of CD40 and CD80 on pRNA-stimulated DC (Figures 2C,D). pRNA alone or in combination with ROT and AA did not affect cell viability (Supplementary Figure 1C). ROT or AA did not affect TNFα secretion (Figure 2E) and upregulation of CD40 and CD80 on LPS-stimulated DC (Figures 2F,G). Collectively, these data indicate pRNA-stimulated DC maturation requires OXPHOS but not glycolysis.

### pRNA-Induced Dendritic Cell Maturation Requires Fatty Acid Oxidation (FAO)

**Figure 3:**
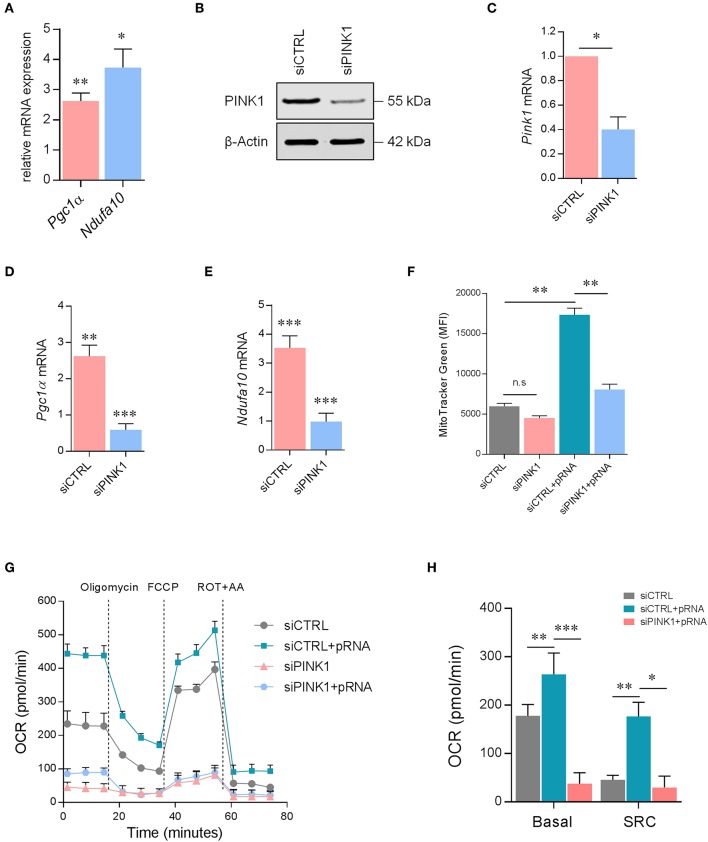
PINK1 is required for OXPHOS in pRNA-stimulated DC. **(A)** Relative mRNA levels were analyzed after 12 h of pRNA stimulation by (qPCR) and normalized to β-actin expression by using the 2^ΔΔ*CT*^ method. Data represents Mean ± SEM of three independent experiments performed in triplicate. ^*^p < 0.05; ^**^p < 0.01 (two-way ANOVA). **(B)** DCs transfected with a control siRNA (siCTRL) and pool of two PINK1-targeting siRNAs. Expression of PINK1 was determined by western blotting. β-actin was used as a loading control. **(C)** *PINK1* mRNA levels were analyzed by qPCR and normalized to β-actin expression by using the 2^ΔΔ*CT*^ method. Data represents Mean ± SEM of three independent experiments performed in triplicate. ^*^p < 0.05 (Student's *t*-test). **(D,E)** mRNA levels were analyzed after 12 h of pRNA stimulation by (qPCR) and normalized to β-actin expression by using the 2^ΔΔ*CT*^ method. Data represents Mean ± SEM of three independent experiments performed in triplicate. ^**^p < 0.01; ^***^p < 0.001 (Student's *t*-test). **(F)** Mean fluorescence intensity of DCs stained with MitoTracker Green^FM^ after 12 h of pRNA stimulation. Data represents Mean ± SEM of three independent experiments performed in triplicate. ^**^p < 0.01 (Student's *t*-test). **(G)** Mitochondrial fitness test of DCs stimulated with pRNA for 12 h. Data represents mean ± SEM of three independent experiments performed in triplicate. **(H)** Data was collected within same experiments as **(G)**, but is shown separately for better understanding. Data represents mean ± SEM of three independent experiments performed in triplicate. ^*^p < 0.05; ^**^p < 0.01; ^***^p < 0.001 (Student's *t*-test).

To unravel the mechanism driving OXPHOS-mediated DC maturation after pRNA stimulation, *NdufA10* and *Pgc1*α gene expression was determined. Peroxisome proliferator-activated receptor gamma co-activator 1-alpha (PGC1α) controls mitochondrial biogenesis (36–38). NdufA10 protein is a subunit of OXPHOS complex I (39). pRNA stimulation of DCs significantly increased *Pgc1*α and *NdufA10* expression, pointing to an increased mitochondrial biogenesis and OXPHOS activity (Figure 3A). Indeed, pRNA stimulation significantly increased mitochondrial content in DC (Figure 3F).

A critical regulator of OXPHOS and mitochondrial homeostasis is PINK1 (40–42). Effective silencing of PINK1 with siRNA (Figures 3B,C) significantly reduced *Pgc1*α (Figure 3D) and *NdufA10* in pRNA-stimulated DC (Figure 3E). As expected, based on the decrease of *Pgc1*α, the mitochondrial content was lower in PINK1 deficient- compared to PINK1 proficient-DC (Figure 3F). Furthermore, the mitochondrial stress test showed that PINK1-silencing significantly reduced basal OCR, SRC (Figures 3G,H), and ATP-linked OCR (Supplementary Figure 1D) in both unstimulated- and pRNA-stimulated DC. Together, these findings indicate that PINK1 is required for pRNA-induced OXPHOS in DC maturation.

**Figure 4:**
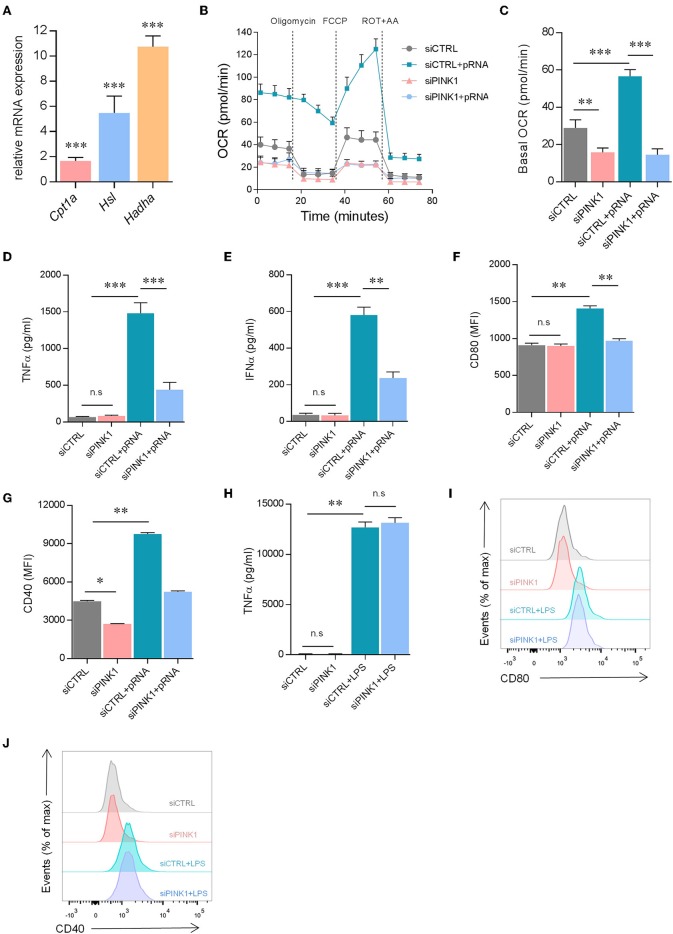
pRNA-stimulated DC maturation requires PINK1-dependent FAO. **(A)** Relative mRNA levels were analyzed after 12 h of pRNA stimulation by (qPCR) and normalized to β-actin expression by using the 2^ΔΔ*CT*^ method. Data represents Mean ± SEM of three independent experiments performed in triplicate. ^***^p < 0.001 (two-way ANOVA). **(B)** OCR was measured in DCs cultured in substrate-limited medium to indicate oxidation of endogenous fatty acids. Quantification of total FAO as the difference between conditions with or without Etomoxir treatment is shown. Data represents mean ± SEM of three independent experiments two-way ANOVA. **(C)** Data was collected within same experiments as **(B)**, but is shown separately for better understanding. Data represents mean ± SEM of three independent experiments two-way ANOVA. ^**^p < 0.01; ^***^p < 0.001 (Student's *t*-test). **(D)** TNFα levels on protein level were measured in the supernatant of the DCs stimulated with pRNA for 12 h. Data represents mean ± SEM of three independent experiments performed in triplicate. ^***^p < 0.001 (Student's *t*-test). **(E)** IFNα levels on protein level were measured in the supernatant of the DCs stimulated with pRNA for 12 h. Data represents mean ± SEM of three independent experiments performed in triplicate. ^**^p < 0.01; ^***^p < 0.001 (Student's *t*-test). **(F)** Mean fluorescence intensity of CD80 in DCs stimulated for 12 h with pRNA. Data represents mean ± SEM of three independent experiments performed in triplicate. ^**^p < 0.01; ^***^p < 0.001 (Student's *t*-test). **(G)** Mean fluorescence intensity of CD40 in DCs stimulated for 12 h with pRNA. Data represents mean ± SEM of three independent experiments performed in triplicate. ^*^p < 0.05; ^**^p < 0.01; (Student's *t*-test). **(H)** TNFα levels on protein level were measured in the supernatant of the DCs stimulated with LPS (100 ng/mL) for 12 h. Data represents mean ± SEM of three independent experiments performed in triplicate. ^**^p < 0.01 (Student's *t*-test). **(I)** Expression of CD80 on DCs stimulated with LPS (100 ng/mL) for 12 h. One experiment of 3 is shown. **(J)** Expression of CD40 on DCs stimulated with LPS (100 ng/mL) for 12 h. One experiment of 3 is shown.

To determine how OXPHOS is increased in pRNA-stimulated DC we investigated the involvement of FAO. Previously, FAO-supplemented OXPHOS was shown in TLR9 stimulated plasmacytoid DCs (pDCs) (43). Here, we show that pRNA stimulation significantly increased expression of *Cpt1*α, *Hadh*α and *Hsl* in DC (Figure 4A). These genes encode enzymes for FAO regulation. A significant increase in FAO was observed in pRNA-stimulated DC (Figures 4B,C; Supplementary Figure 1E). PINK1 silencing reduced FAO in both unstimulated- and pRNA-stimulated DC (Figures 4B,C; Supplementary Figure 1E). Collectively, these data indicate that FAO is involved in OXPHOS increase in pRNA-stimulated DC.

To investigate whether pRNA-stimulated DC maturation requires PINK1 regulated metabolic changes, the secretion of pro-inflammatory cytokine TNFα and IFNα and membrane expression of costimulatory molecules CD40 and CD80 were assessed. Upon pRNA stimulation, PINK1 knockdown DC were viable (cell viability over 97%, Supplementary Figure 2A) but produced significantly less TNFα and IFNα compared to PINK1 proficient cells (Figures 4D,E). Moreover, increased expression of CD40 and CD80 following pRNA stimulation was also significantly inhibited by PINK1 knockdown (Figures 4F,G). However, PINK1 knockdown did not affect LPS stimulated TNFα secretion (Figure 4H) or expression of CD80 and CD40 (Figures 4I,J). Together, this indicates that pRNA-mediated DC maturation is dependent on PINK1-regulated FAO.

### PINK1-Mediated Phosphorylation of BCKDE1α Is Required for pRNA-Induced Maturation of Dendritic Cells

**Figure 5:**
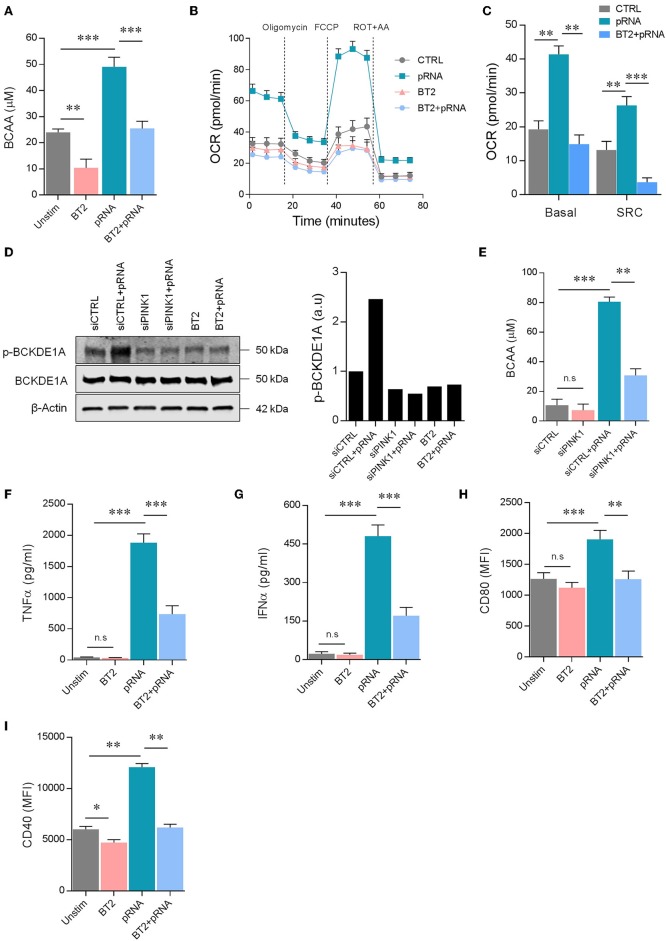
PINK1 regulated BCAAs induce FAO in pRNA-stimulated DC. **(A)** Total BCAA concentrations were measured in the DCs stimulated for 12 h with pRNA in the presence or absence of 50 μM BT2, using a colorimetric assay kit. Data represents Mean ± SEM of three independent experiments performed in triplicate. ^**^p < 0.01; ^***^p < 0.001 (Student's *t*-test). **(B)** OCR was measured in DC cultured in substrate-limited medium in the presence or absence of 50 μM BT2. Quantification of total FAO as the difference between conditions with or without Etomoxir treatment is shown. Data represents mean ± SEM of three independent experiments performed in triplicate. **(C)** Data was collected within same experiments as **(B)**, but is shown separately for better understanding. Data represents mean ± SEM of three independent experiments performed in triplicate. ^**^p < 0.01; ^***^p < 0.001 (Student's *t*-test). **(D)** Protein levels of phosphorylated p-BCKDE1α and total BCKDE1α in DCs stimulated for 12 h with pRNA under indicated conditions. β-actin was used as a loading control. **(E)** Total BCAA concentrations were measured in siControl or siPINK1 transfected DCs using a colorimetric assay kit. Data represents Mean ± SEM of three independent experiments performed in triplicate. ^**^p < 0.01; ^***^p < 0.001 (Student's *t*-test). **(F)** TNFα levels on protein level were measured in the supernatant of the DCs stimulated for 12 h with pRNA in the presence or absence of 50 μM BT2. Data represents mean ± SEM of three independent experiments performed in triplicate. ^***^p < 0.001 (Student's *t*-test). **(G)** IFNα levels on protein level were measured in the supernatant of the DCs stimulated for 12 h with pRNA in the presence or absence of 50 μM BT2. Data represents mean ± SEM of three independent experiments performed in triplicate. ^***^p < 0.001 (Student's *t*-test). **(H)** Mean fluorescence intensity of CD80 in DCs stimulated for 12 h with pRNA in the presence or absence of 50 μM BT2. Data represents mean ± SEM of three independent experiments performed in triplicate. ^**^p < 0.01; ^***^p < 0.001 (Student's *t*-test). **(I)** Mean fluorescence intensity of CD40 in DCs stimulated for 12 h with pRNA in the presence or absence of 50 μM BT2. Data represents mean ± SEM of three independent experiments performed in triplicate. ^*^p < 0.05; ^**^p < 0.01 (Student's *t*-test).

pRNA-mediated TLR stimulation promotes FAO in DCs. *Pgc1*α expression and FAO is increased by BCAA in myotubes (44). We hypothesize that TLR stimulation increases BCAA levels in DCs, leading to enhanced FAO. Indeed, pRNA stimulation significantly increased BCAA levels in DC (Figure 5A). The homeostasis of BCAA is critically regulated by branched-chain α-ketoacid dehydrogenase (BCKDH) complex, which oxidatively decarboxylates branch-chain α-ketoacids (BCKA) from BCAA. BCKDH is regulated by phosphorylation and dephosphorylation events. BCKDH kinase inactivates the complex by phosphorylation of the E1α subunit of the complex, while BCKDH phosphatase activates the complex by dephosphorylation of E1α. BCKDH kinase is crucial in the regulation of BCKDH activity (45). Modulation of BCKDH kinase activity changes BCAA homeostasis. 3,6-Dichloro-benzo[b]thiophene-2-carboxylic acid (BT2) is an inhibitor of BCKDH kinase (46). Here, inhibitor BT2 significantly reduced basal and pRNA stimulation-induced BCAA levels in DCs (Figure 5A), indicating involvement of BCKDH kinase. Importantly, BT2 significantly reduced pRNA-stimulated FAO in DC (Figures 5B,C; Supplementary Figure 1F).

We here show that PINK1-dependent FAO requires BCAA. PINK1 phosphorylates several proteins (11, 40, 47–50) and might therefore also regulate BCKDH. Indeed, pRNA stimulation increased phopshorylation of BCKDH subunit BCKDE1α in DCs, which could be inhibited by BCKDH kinase inhibitor BT2 (Figure 5D). Knockdown of PINK1 or addition of BT2 reduced phopshorylation of BCKDE1α, and thereby the BCAA levels were diminished in DCs and no increase was observed after pRNA stimulation (Figure 5D). Knockdown of PINK1 or addition of BT2 reduced TNFα and IFNα production and expression of CD40 and CD80 by pRNA-stimulated DCs (Figures 4D–G, 5E–I). Collectively, these data show that DC maturation requires kinase activity of PINK1 for BCKDE1α phosphorylation. Phosphorylation of BCKDE1α disrupts BCAA catabolism and thereby promotes FAO, resulting in maturation of DC after pRNA stimulation.

### PINK1 Kinase Activity Determines Immune Stimulatory Capacity of Human DC

**Figure 6:**
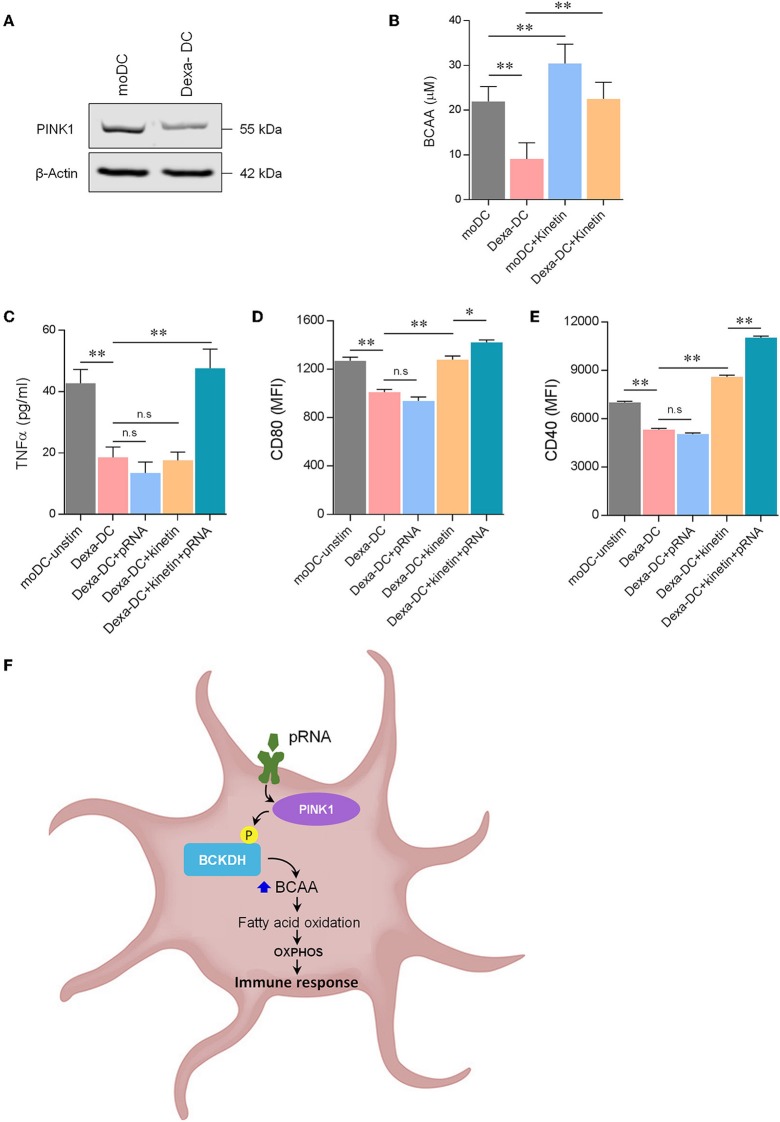
Kinetin primes immunosuppressive DC for maturation. **(A)** Expression of PINK1 in DCs was determined by western blotting. β-actin was used as a loading control. **(B)** Total BCAA concentrations were measured in DCs, stimulated for 12 h with pRNA in the presence or absence of 20 μM kinetin, using a colorimetric assay kit. Data represents Mean ± SEM of three independent experiments performed in triplicate. ^**^p < 0.01 (Student's *t*-test). **(C)** TNFα levels on protein level were measured in the supernatant of the DCs stimulated for 12 h with pRNA in the presence or absence of 20 μM kinetin. Data represents mean ± SEM of three independent experiments performed in triplicate. ^**^p < 0.01 (Student's *t*-test). **(D)** Mean fluorescence intensity of CD80 in DCs stimulated for 12 h with pRNA in the presence or absence of 20 μM kinetin. Data represents mean ± SEM of three independent experiments performed in triplicate. ^*^p < 0.05; ^**^p < 0.01 (Student's *t*-test). **(E)** Mean fluorescence intensity of CD40 in DCs stimulated for 12 h with pRNA in the presence or absence of 20 μM kinetin. Data represents mean ± SEM of three independent experiments performed in triplicate. ^**^p < 0.01 (Student's *t*-test). **(F)** Mechanistic model of OXPHOS-mediated DC activation. Activation of TLR7/8 with pRNA lead to increase in OXPHOS. pRNA stimulation phosphorylates BCKDE1α in a PINK1-dependent manner. pRNA stimulation increased branched-chain amino acid (BCAA) to promote FAO. Increased FAO and OXPHOS were required for DC activation.

Various factors can transform immune-stimulatory DCs into immune-suppressive DCs, characterized by low expression of co-stimulatory molecules and altered cytokine production (51). Here, we generated immunosuppressive DCs by adding dexamethasone during culture (Dexa-DC). Indeed, the expression of co-stimulatory molecules i.e., CD40 and CD80 and the production of TNFα was low in Dexa-DC and this could not be increased by pRNA stimulation (Figures 6D,E; Supplementary Figure 1H). Moreover, Dexa-DC had lower PINK1 expression (Figure 6A) and lower levels of BCAA compared to DCs (Figure 6B). Kinetin, a membrane permeable precursor of ATP analog N^6^ furfuryl ATP (KTP), increases kinase activity of PINK1 (52). Here, kinetin significantly increased BCAA levels and CD40 expression but not CD80 expression in Dexa-DCs (Figures 6B,D,E). pRNA in combination with kinetin significantly increased CD40 and CD80 expression and the production of TNFα in Dexa-DC (Figures 6C–E). Together, these data show that an increase in PINK1 kinase activity restored the immune- stimulatory capacity of immune-suppressive Dexa-DC.

## Discussion

Pathogen recognition by antigen presenting cells to initiate immune responses is a key process in host defense. For *in vitro* studies, TLR4 ligand LPS is most commonly used to mimic inflammatory responses. LPS promotes metabolic rewiring resembling the Warburg effect in both DCs and bone marrow-derived macrophages (BMDMs) (53, 54). While knowledge concerning molecular pathways controlling metabolic routes in DC is accumulating (55), the specificity of metabolic adaptations after stimulation with different TLR ligands is not studied in detail. Here, we demonstrate that stimulation of DC via TLR7/8 engages a different metabolic pathway than DC stimulated *via* TLR4. We here confirm previous results concerning LPS stimulation of DC resulting in a switch to glycolysis for maturation. In contrast, TLR7/8 stimulated DC increase OXPHOS concurrently with glycolysis. However, this glycolysis diminishes after 12 h while increased OXPHOS is maintained. A recent study demonstrated that different DC subtypes, i.e., human pDCs and monocyte-derived DCs (moDCs), have distinct metabolic requirements for maturation. Upon stimulation of RIG-I, moDC increase glycolysis whereas pDCSs employ OXPHOS (56). Previously, we reported that human blood-derived DCs i.e., cDC2^+^ mDC and pDC for maturation switch to glycolysis and OXPHOS, respectively (25). This underlines the distinct metabolic adaptations triggered by different pathogens depending on their specific TLR binding.

Specific stimulation of DC via TLR7/8 by pRNA increased OXPHOS accompanied by an increased mitochondrial content. A critical regulator of OXPHOS and mitochondrial homeostasis is PINK1. Indeed, PINK1 knockdown reduced the pRNA-induced increase of OXPHOS and mitochondrial content. PINK1 is also involved in mitophagy and has a role in MHC antigen presentation (57–59). Lack of accumulation of mitochondrial content in PINK1 deficient cells upon pRNA stimulation indicates absence of mitophagy in our model (60). Albeit, our data show that PINK1 is crucial for pRNA-stimulated DC maturation, indicating a mitophagy-independent function of PINK1. Previously, PINK1 is described to be crucial for survival (61, 62). However, the viability of PINK1 knockdown DC was not affected either in the presence nor absence of pRNA (Supplementary Information). Furthermore, it is shown that PINK1 deficiency results in less ATP due to impaired mitochondrial fission leading to defective assembly of the ETC complexes, and reduction in mitochondrial respiration and mitochondrial buffering capacity (63–66). Total intracellular ATP levels were increased in both pRNA maturation of DC and PINK1-knockdown DC (Supplementary Information). We here show that PINK1 knockdown DC maintain cell viability by reprogramming bioenergetics from OXPHOS to glycolysis (Supplementary Information). Loss of PINK1 has been reported to result in stabilization of HIF1α which stimulates glycolysis (67). Indeed, glucose uptake was increased in PINK1-deficient cells and further increased by pRNA stimulation (Supplementary Information). Intriguingly, *Glut1* levels did not change upon pRNA stimulation, however loss of PINK1 increased *Glut1* levels, which has been reported to be involved in glucose uptake in DCs (68). These data suggest that loss of PINK1 reprograms glucose metabolism (Supplementary Information), possibly through HIF1α, in DCs to sustain cell viability.

We here identified PINK1 as a novel regulator of BCAAs, which are important for FAO to supplement OXPHOS crucial for TLR7/8-induced DC maturation. The activity of BCKDH complex is regulated *via* phosphorylation/dephosphorylation of BCKDE1α. Previously, PINK1 is shown to be able to phosphorylate target proteins in a kinase-dependent manner (11, 40, 47–50, 69). Our data show that PINK1 regulates phosphorylation of BCKDE1α in DCs and thus is a critical regulator of BCKDH complex for BCAA catabolism. Interestingly, Parkinson disease patients, who have mutated PINK1 in neurons (64), have decreased BCAA serum levels associated with an increase in the clinical severity of the disease (70). Therefore, our finding that PINK1 regulates BCAA catabolism may have important implications for patients suffering from Parkinson disease. TLR7/8 stimulation with pRNA increased BCAA promoting FAO in DC. Similarly, for pDC maturation, TLR9 agonist, CpG-A, also enhanced FAO (43). In contrast, FAO is not necessary for maturation of mouse bone marrow-derived DCs stimulated with LPS (7). Collectively, DCs engage distinct metabolic routes for maturation upon stimulation *via* different TLRs.

Our data identified PINK1 as a critical regulator of BCAA catabolism, whereas previously PINK1 is shown to critically regulate FAO (71). This led to the question of whether PINK1 *via* BCAAs contribute to FAO. We observed that PINK1 knockdown reduced pRNA-induced FAO in DCs. BCAAs are required for fatty acid synthesis (72) and promote FAO in muscles and myotubes (44, 73). Consistently, inhibition of BCAAs levels abrogated pRNA-induced FAO in DC. BCAA leucine increases expression of *Cpt1, Cpt2* (74), *PPAR***γ** (75), *Pgc1*α, and *Nrf-1* (76). Indeed, *Pgc1*α expression and mitochondrial content were increased after pRNA stimulation of DC, hinting at increased FAO. Another possible mechanism for BCAA-mediated FAO is that leucine increases SIRT1 activity, which in turn phosphorylates AMPK (76). AMPK subsequently regulates FAO through phosphorylation of acetyl-CoA carboxylase 2 (77). Given the increase in BCAA after TLR7/8 stimulation of DC by pRNA it seems likely that SIRT1-phospho AMPK increases FAO. An alternative mechanism for BCAA-mediated FAO could be that leucine inhibits mTOR (78), which is central to lipid homoeostasis and mTOR inhibition increases FAO (79). Therefore, it is possible that pRNA stimulation of DC increased BCAA and thereby inhibited mTOR resulting in FAO.

In immunosuppressive DC (Dexa-DC), PINK1 levels were diminished and increasing PINK1 kinase activity directed the immunosuppressive phenotype toward an immunostimulatory phenotype. Previously, Ibrahim et al. showed that DC with lower lipid content are immunosuppressive (80). PINK1 expression is indeed necessary for regulating lipid droplet formation and mitochondrial FAO (81). Activation of DC induces fatty acid synthesis, which results in increased lipid storage in lipid droplets (82). Together, these reports suggest that lower PINK1 levels likely result in reduced fatty acid synthesis and lipid storage in immunosuppressive DC. Induction of PINK1 kinase activity likely increased fatty acid synthesis *via* BCAAs in immunosuppressive DC, which was then utilized for FAO upon TLR7/8 stimulation. The observation that PINK1 levels were lower in immunosuppressive DC is in contrast with the observed effects in Parkinson patients and *Pink1*^−/−^ mice. There, high levels of TNF-α, IFN-γ, and IL-6 are all indicative of an inflammatory phenotype that are observed (83, 84). This can be due to reduced OXPHOS activity, mitophagy induction, and higher oxidized mtDNA, acting as an inflammatory signal in the Parkinson model (84, 85). Here, we observed an increase in OXPHOS activity and did not observe mitophagy possibly explaining the immune suppressive phenotype.

Our data, together with recent reports, show that distinct metabolic programs in DCs can be induced dependent on the stimulus. Manipulation of human DC functionality via metabolic programming needs a broader understanding, especially since the diversity of responses induced by different PRRs is very broad. We identified the PINK1-BCAAs axis as a novel metabolic programming pathway in DCs (Figure 6F), hence, strategies aiming at modifying this axis might provide a clinical benefit in immune-related pathologies.

## Data Availability Statement

All datasets generated for this study are included in the manuscript/Supplementary Files.

## Author Contributions

FB and IV conceived the research and wrote the manuscript. FB performed the experiments and analyzed the data. IV supervised the research.

### Conflict of Interest

The authors declare that the research was conducted in the absence of any commercial or financial relationships that could be construed as a potential conflict of interest.

## References

[1] Tel J Anguille S Waterborg CE Smits EL Figdor CG de Vries IJ. Tumoricidal activity of human dendritic cells. Trends Immunol. (2014) 35:38–46. 10.1016/j.it.2013.10.007 24262387 PMC7106406

[2] Hemmi H Akira S. TLR signalling and the function of dendritic cells. Chem Immunol Allergy. (2005) 86:120–35. 10.1159/000086657 15976491

[3] Mishra A. Metabolic plasticity in dendritic cell responses: implications in allergic asthma. J Immunol Res. (2017) 2017:5134760. 10.1155/2017/5134760 29387732 PMC5745769

[4] Amiel E Everts B Fritz D Beauchamp S Ge B Pearce E. Mechanistic target of rapamycin inhibition extends cellular lifespan in dendritic cells by preserving mitochondrial function. J Immunol. (2014) 193:2821–30. 10.4049/jimmunol.1302498 25108022 PMC4302759

[5] Krawczyk CM Holowka T Sun J Blagih J Amiel E DeBerardinis R. Toll-like receptor-induced changes in glycolytic metabolism regulate dendritic cell activation. Blood. (2010) 115:4742–9. 10.1182/blood-2009-10-249540 20351312 PMC2890190

[6] Thwe PM Pelgrom L Cooper R Beauchamp S Reisz JA D'Alessandro A. Cell-intrinsic glycogen metabolism supports early glycolytic reprogramming required for dendritic cell immune responses. Cell Metab. (2017) 26:558–67.e5. 10.1016/j.cmet.2017.08.012 28877459 PMC5657596

[7] Everts B Amiel E Huang SC Smith AM Lam WY. TLR-driven early glycolytic reprogramming via the kinases TBK1-IKKvarepsilon supports the anabolic demands of dendritic cell activation. Nat Immunol. (2014) 15:323–32. 10.1038/ni.2833 24562310 PMC4358322

[8] Bajwa G DeBerardinis RJ Shao B Hall B Farrar J Gill MA. Cutting edge: critical role of glycolysis in human plasmacytoid dendritic cell antiviral responses. J Immunol. (2016) 196:2004–9. 10.4049/jimmunol.1501557 26826244 PMC4761472

[9] Szatmari I Torocsik D Agostini M Nagy T Gurnell M Barta E. PPARgamma regulates the function of human dendritic cells primarily by altering lipid metabolism. Blood. (2007) 110:3271–80. 10.1182/blood-2007-06-096222 17664351

[10] Ferreira GB Vanherwegen AS Eelen G Gutierrez AC Van Lommel L Marchal K. Vitamin D3 induces tolerance in human dendritic cells by activation of intracellular metabolic pathways. Cell Rep. (2015) 10:711–25. 10.1016/j.celrep.2015.01.013 25660022

[11] Weihofen A Thomas KJ Ostaszewski BL Cookson M Selkoe DJ. Pink1 forms a multiprotein complex with Miro and Milton, linking Pink1 function to mitochondrial trafficking. Biochemistry. (2009) 48:2045–52. 10.1021/bi8019178 19152501 PMC2693257

[12] Zhou C Huang Y Shao Y May J Prou D Perier C. The kinase domain of mitochondrial PINK1 faces the cytoplasm. Proc Natl Acad Sci USA. (2008) 105:12022–7. 10.1073/pnas.0802814105 18687899 PMC2575334

[13] Voigt A Berlemann LA Winklhofer KF. The mitochondrial kinase PINK1: functions beyond mitophagy. J Neurochem. (2016) 139(Suppl 1):232–9. 10.1111/jnc.13655 27251035

[14] Whitworth AJ Lee JR Ho VM Flick R Chowdhury R McQuibban GA Rhomboid-7 and HtrA2/Omi act in a common pathway with the Parkinsons disease factors Pink1 and Parkin. Dis Model Mech. (2008) 1:168–74. 10.1242/dmm.000109 19048081 PMC2562193

[15] Jin SM Lazarou M Wang C Kane LA Narendra D Youle RJ. Mitochondrial membrane potential regulates PINK1 import and proteolytic destabilization by PARL. J Cell Biol. (2010) 191:933–42. 10.1083/jcb.201008084 21115803 PMC2995166

[16] Deas E Plun-Favreau H Gandhi S Desmond H Kjaer S Loh S. PINK1 cleavage at position A103 by the mitochondrial protease PARL. Hum Mol Genet. (2011) 20:867–79. 10.1093/hmg/ddq526 21138942 PMC3033179

[17] Meissner C Lorenz H Weihofen A Selkoe DJ Lemberg M. The mitochondrial intramembrane protease PARL cleaves human Pink1 to regulate Pink1 trafficking. J Neurochem. (2011) 117:856–67. 10.1111/j.1471-4159.2011.07253.x 21426348

[18] Shi G Lee JR Grimes DA Racacho L Ye D Yang H Functional alteration of PARL contributes to mitochondrial dysregulation in Parkinsons disease. Hum Mol Genet. (2011) 20:1966–74. 10.1093/hmg/ddr077 21355049

[19] Greene AW Grenier K Aguileta MA Muise S Farazifard R Haque ME. Mitochondrial processing peptidase regulates PINK1 processing, import and Parkin recruitment. EMBO Rep. (2012) 13:378–385. 10.1038/embor.2012.14 22354088 PMC3321149

[20] Yamano K Youle RJ. PINK1 is degraded through the N-end rule pathway. Autophagy. (2013) 9:1758–69. 10.4161/auto.24633 24121706 PMC4028335

[21] McLelland GL Soubannier V Chen CX McBride HM Fon EA. Parkin and PINK1 function in a vesicular trafficking pathway regulating mitochondrial quality control. EMBO J. (2014) 33:282–95. 10.1002/embj.201385902 24446486 PMC3989637

[22] Grohmann U Mondanelli G Belladonna ML Orabona C Pallotta M Iacono A. Amino-acid sensing and degrading pathways in immune regulation. Cytokine Growth Factor Rev. (2017) 35:37–45. 10.1016/j.cytogfr.2017.05.004 28545736

[23] Li P Yin YL Li D Kim SW Wu G. Amino acids and immune function. Br J Nutr. (2007) 98:237–252. 10.1017/S000711450769936X 17403271

[24] Johnson MO Wolf MM Madden MZ Andrejeva G Sugiura A Contreras DC. Distinct regulation of Th17 and Th1 cell differentiation by glutaminase-dependent metabolism. Cell. (2018) 175:1780–95.e19. 10.1016/j.cell.2018.10.001 30392958 PMC6361668

[25] Basit F Mathan T Sancho D de Vries IJM. Human dendritic cell subsets undergo distinct metabolic reprogramming for immune response. Front Immunol. (2018) 9:2489. 10.3389/fimmu.2018.02489 30455688 PMC6230993

[26] Cruzat V Macedo Rogero M Noel Keane K Curi R Newsholme P. Glutamine: metabolism and immune function, supplementation and clinical translation. Nutrients. (2018) 10:E1564. 10.20944/preprints201809.0459.v1 30360490 PMC6266414

[27] Kim SH Roszik J Grimm EA Ekmekcioglu S. Impact of l-arginine metabolism on immune response and anticancer immunotherapy. Front Oncol. (2018) 8:67. 10.3389/fonc.2018.00067 29616189 PMC5864849

[28] Popovic PJ Zeh HJ 3rd Ochoa JB. Arginine and immunity. J Nutr. (2007) 137:1681S–6S. 10.1093/jn/137.6.1681S 17513447

[29] Grimble RF. The effects of sulfur amino acid intake on immune function in humans. J Nutr. (2006) 136:1660S–5S. 10.1093/jn/136.6.1660S 16702336

[30] Lerin C Goldfine AB Boes T Liu M Kasif S Dreyfuss JM. Defects in muscle branched-chain amino acid oxidation contribute to impaired lipid metabolism. Mol Metab. (2016) 5:926–36. 10.1016/j.molmet.2016.08.001 27689005 PMC5034611

[31] de Vries IJ Eggert AA Scharenborg NM Vissers J Lesterhuis WJ. Phenotypical and functional characterization of clinical grade dendritic cells. J Immunother. (2002) 25:429–38. 10.1097/00002371-200209000-00007 12218781

[32] Pelgrom LR van der Ham AJ Everts B. Analysis of TLR-induced metabolic changes in dendritic cells using the seahorse XF(e)96 extracellular flux analyzer. Methods Mol Biol. (2016) 1390:273–85. 10.1007/978-1-4939-3335-8_17 26803635

[33] Pike LS Smift AL Croteau NJ Ferrick D Wu M. Inhibition of fatty acid oxidation by etomoxir impairs NADPH production and increases reactive oxygen species resulting in ATP depletion and cell death in human glioblastoma cells. Biochim Biophys Acta. (2011) 1807:726–34. 10.1016/j.bbabio.2010.10.022 21692241

[34] Tu LN Zhao AH Hussein M Stocco DM Selvaraj V. Translocator protein (TSPO) affects mitochondrial fatty acid oxidation in steroidogenic cells. Endocrinology. (2016) 157:1110–21. 10.1210/en.2015-1795 26741196 PMC4769361

[35] Yang M Chadwick AE Dart C Kamishima T Quayle J. Bioenergetic profile of human coronary artery smooth muscle cells and effect of metabolic intervention. PLoS ONE. (2017) 12:e0177951. 10.1371/journal.pone.0177951 28542339 PMC5438125

[36] Austin S St-Pierre J. PGC1alpha and mitochondrial metabolism–emerging concepts and relevance in ageing and neurodegenerative disorders. J Cell Sci. (2012) 125:4963–71. 10.1242/jcs.113662 23277535

[37] Uguccioni G Hood DA. The importance of PGC-1alpha in contractile activity-induced mitochondrial adaptations. Am J Physiol Endocrinol Metab. (2011) 300:E361–71. 10.1152/ajpendo.00292.2010 21081705

[38] Fernandez-Marcos PJ Auwerx J. Regulation of PGC-1alpha, a nodal regulator of mitochondrial biogenesis. Am J Clin Nutr. (2011) 93:884S–90. 10.3945/ajcn.110.001917 21289221 PMC3057551

[39] Hoefs SJ van Spronsen FJ Lenssen EW Nijtmans LG Rodenburg RJ Smeitink JA. NDUFA10 mutations cause complex I deficiency in a patient with Leigh disease. (2011) Eur J Hum Genet. 19:270–4. 10.1038/ejhg.2010.204 21150889 PMC3061993

[40] Morais VA Haddad D Craessaerts K De Bock PJ Swerts J Vilain S. PINK1 loss-of-function mutations affect mitochondrial complex I activity via NdufA10 ubiquinone uncoupling. Science. (2014) 344:203–7. 10.1126/science.1249161 24652937

[41] Bueno M Lai YC Romero Y Brands St J Croix C Kamga C. PINK1 deficiency impairs mitochondrial homeostasis and promotes lung fibrosis. J Clin Invest. (2015) 125:521–38. 10.1172/JCI74942 25562319 PMC4319413

[42] Heeman B Van den Haute C Aelvoet SA Valsecchi F Rodenburg RJ Reumers V. Depletion of PINK1 affects mitochondrial metabolism, calcium homeostasis and energy maintenance. J Cell Sci. (2011) 124:1115–25. 10.1242/jcs.078303 21385841

[43] Wu D Sanin DE Everts B Chen Q Qiu J Buck MD. Type 1 interferons induce changes in core metabolism that are critical for immune function. Immunity. (2016) 44:1325–36. 10.1016/j.immuni.2016.06.006 27332732 PMC5695232

[44] Liang C Curry BJ Brown PL Zemel M. Leucine modulates mitochondrial biogenesis and SIRT1-AMPK signaling in C2C12 myotubes. J Nutr Metab. (2014) 2014:239750. 10.1155/2014/239750 25400942 PMC4220583

[45] Wynn RM Kato M Machius M Chuang JL Li J Tomchick DR. Molecular mechanism for regulation of the human mitochondrial branched-chain alpha-ketoacid dehydrogenase complex by phosphorylation. Structure. (2004) 12:2185–96. 10.1016/j.str.2004.09.013 15576032

[46] Tso SC Gui WJ Wu CY Chuang JL Qi X Skvora KJ. Benzothiophene carboxylate derivatives as novel allosteric inhibitors of branched-chain alpha-ketoacid dehydrogenase kinase. J Biol Chem. (2014) 289:20583–93. 10.1074/jbc.M114.569251 24895126 PMC4110271

[47] Pridgeon JW Olzmann JA Chin LS Li L. PINK1 protects against oxidative stress by phosphorylating mitochondrial chaperone TRAP1. PLoS Biol. (2007) 5:e172. 10.1371/journal.pbio.0050172 17579517 PMC1892574

[48] Plun-Favreau H Klupsch K Moisoi N Gandhi S Kjaer S Frith D The mitochondrial protease HtrA2 is regulated by Parkinsons disease-associated kinase PINK1. Nat Cell Biol. (2007) 9:1243–52. 10.1038/ncb1644 17906618

[49] Chen Y Dorn GW 2nd. PINK1-phosphorylated mitofusin 2 is a Parkin receptor for culling damaged mitochondria. Science. (2013) 340:471–5. 10.1126/science.1231031 23620051 PMC3774525

[50] Arena G Gelmetti V Torosantucci L Vignone D Lamorte G De Rosa P. PINK1 protects against cell death induced by mitochondrial depolarization, by phosphorylating Bcl-xL and impairing its pro-apoptotic cleavage. Cell Death Differ. (2013) 20:920–30. 10.1038/cdd.2013.19 23519076 PMC3679455

[51] Domogalla MP Rostan PV Raker VK Steinbrink K. Tolerance through education: how tolerogenic dendritic cells shape immunity. Front Immunol. (2017) 8:1764. 10.3389/fimmu.2017.01764 29375543 PMC5770648

[52] Hertz NT Berthet A Sos ML Thorn KS Burlingame AL Nakamura K. A neo-substrate that amplifies catalytic activity of parkinsons-disease-related kinase PINK1. Cell. (2013) 154:737–47. 10.1016/j.cell.2013.07.030 23953109 PMC3950538

[53] Pearce EJ Everts B. Dendritic cell metabolism. Nat Rev Immunol. (2015) 15:18–29. 10.1038/nri3771 25534620 PMC4495583

[54] ONeill LA A critical role for citrate metabolism in LPS signalling. Biochem J. (2011) 438:e5–6. 10.1042/BJ20111386 21867483

[55] Wculek S Sofía Khouili C Elena P Heras-Murillo I David S. Metabolic control of dendritic cell functions: digesting information. Front. Immunol. (2019) 10:775. 10.3389/fimmu.2019.00775 31073300 PMC6496459

[56] Fekete T Suto MI Bencze D Mazlo A Szabo A Biro T. Human plasmacytoid and monocyte-derived dendritic cells display distinct metabolic profile upon rig-i activation. Front Immunol. (2018) 9:3070. 10.3389/fimmu.2018.03070 30622542 PMC6308321

[57] McWilliams TG Prescott AR Montava-Garriga L Ball G Singh F Barini E. Basal mitophagy occurs independently of PINK1 in mouse tissues of high metabolic demand. Cell Metab. (2018) 27:439–49.e5. 10.1016/j.cmet.2017.12.008 29337137 PMC5807059

[58] Vats D Mukundan L Odegaard JI Zhang L Smith K Morel CR. Oxidative metabolism and PGC-1beta attenuate macrophage-mediated inflammation. Cell Metab. (2006) 4:13–24. 10.1016/j.cmet.2006.05.011 16814729 PMC1904486

[59] Bell C English L Boulais J Chemali M Caron-Lizotte O Desjardins M. Quantitative proteomics reveals the induction of mitophagy in tumor necrosis factor-alpha-activated (TNFalpha) macrophages. Mol Cell Proteomics. (2013) 12:2394–407. 10.1074/mcp.M112.025775 23674617 PMC3769319

[60] Mauro-Lizcano M Esteban-Martinez L Seco E Serrano-Puebla A Garcia-Ledo L Figueiredo-Pereira C. New method to assess mitophagy flux by flow cytometry. Autophagy. (2015) 11:833–43. 10.1080/15548627.2015.1034403 25945953 PMC4509449

[61] Petit A Kawarai T Paitel E Sanjo N Maj M Scheid M. Wild-type PINK1 prevents basal and induced neuronal apoptosis, a protective effect abrogated by Parkinson disease-related mutations. J Biol Chem. (2005) 280:34025–32. 10.1074/jbc.M505143200 16079129

[62] Klinkenberg M Thurow N Gispert S Ricciardi F Eich F Prehn J. Enhanced vulnerability of PARK6 patient skin fibroblasts to apoptosis induced by proteasomal stress. Neuroscience. (2010) 166:422–34. 10.1016/j.neuroscience.2009.12.068 20045449

[63] Liu W Acin-Perez R Geghman KD Manfredi G Lu B Li C. Pink1 regulates the oxidative phosphorylation machinery via mitochondrial fission. Proc Natl Acad Sci USA. (2011) 108:12920–4. 10.1073/pnas.1107332108 21768365 PMC3150934

[64] Morais VA Verstreken P Roethig A Smet J Snellinx A Vanbrabant M Parkinsons disease mutations in PINK1 result in decreased Complex I activity and deficient synaptic function. EMBO Mol Med. (2009) 1:99–111. 10.1002/emmm.200900006 20049710 PMC3378121

[65] Gegg ME Cooper JM Schapira AH Taanman JW. Silencing of PINK1 expression affects mitochondrial DNA and oxidative phosphorylation in dopaminergic cells. PLoS ONE. (2009) 4:e4756. 10.1371/journal.pone.0004756 19270741 PMC2649444

[66] Amo T Sato S Saiki S Wolf AM Toyomizu M Gautier CA Mitochondrial membrane potential decrease caused by loss of PINK1 is not due to proton leak, but to respiratory chain defects. Neurobiol Dis. (2011) 41:111–8. 10.1016/j.nbd.2010.08.027 20817094

[67] Requejo-Aguilar R Lopez-Fabuel I Fernandez E Martins LM Almeida A Bolaños JP. PINK1 deficiency sustains cell proliferation by reprogramming glucose metabolism through HIF1. Nat Commun. (2014) 5:4514. 10.1038/ncomms5514 25058378

[68] Everts B Pearce EJ. Metabolic control of dendritic cell activation and function: recent advances and clinical implications. Front Immunol. (2014) 5:203. 10.3389/fimmu.2014.00203 24847328 PMC4021118

[69] Wang X Winter D Ashrafi G Schlehe J Wong YL Selkoe D. PINK1 and Parkin target Miro for phosphorylation and degradation to arrest mitochondrial motility. Cell. (2011) 147:93–906. 10.1016/j.cell.2011.10.018 22078885 PMC3261796

[70] Tosukhowong P Boonla C Dissayabutra T Kaewwilai L Muensri S Chotipanich C Biochemical and clinical effects of whey protein supplementation in Parkinsons disease: a pilot study. J Neurol Sci. (2016) 367:162–70. 10.1016/j.jns.2016.05.056 27423583

[71] Course MM Scott AI Schoor C Hsieh CH Papakyrikos AM Winter D. Phosphorylation of MCAD selectively rescues PINK1 deficiencies in behavior and metabolism. Mol Biol Cell. (2018) 29:1219–27. 10.1091/mbc.E18-03-0155 29563254 PMC5935071

[72] Crown SB Marze N Antoniewicz MR. Catabolism of branched chain amino acids contributes significantly to synthesis of odd-chain and even-chain fatty acids in 3T3-L1 adipocytes. PLoS ONE. (2015) 10:e0145850. 10.1371/journal.pone.0145850 26710334 PMC4692509

[73] Tang JE Moore DR Kujbida GW Tarnopolsky M Phillips SM. Ingestion of whey hydrolysate, casein, or soy protein isolate: effects on mixed muscle protein synthesis at rest and following resistance exercise in young men. J Appl Physiol. (2009) 107:987–92. 10.1152/japplphysiol.00076.2009 19589961

[74] Rawat AK Korthikunta V Gautam S Pal S Tadigoppula N Tamrakar A. 4-Hydroxyisoleucine improves insulin resistance by promoting mitochondrial biogenesis and act through AMPK and Akt dependent pathway. Fitoterapia. (2014) 99:307–17. 10.1016/j.fitote.2014.10.006 25454462

[75] Schnuck JK Sunderland KL Gannon NP Kuennen M Vaughan RA. Leucine stimulates PPARbeta/delta-dependent mitochondrial biogenesis and oxidative metabolism with enhanced GLUT4 content and glucose uptake in myotubes. Biochimie. (2016) 128–129:1–7. 10.1016/j.biochi.2016.06.009 27345255

[76] Sun X Zemel MB Leucine modulation of mitochondrial mass and oxygen consumption in skeletal muscle cells and adipocytes. Nutr Metab. (2009). 56:26 10.1186/1743-7075-6-26 PMC2701939 19500359

[77] ONeill HM Holloway GP Steinberg GR AMPK regulation of fatty acid metabolism and mitochondrial biogenesis: implications for obesity. Mol Cell Endocrinol. (2013) 366:135–51. 10.1016/j.mce.2012.06.019 22750049

[78] Haruta T Uno T Kawahara J Takano A Egawa K Sharma P. A rapamycin-sensitive pathway down-regulates insulin signaling via phosphorylation and proteasomal degradation of insulin receptor substrate-1. Mol Endocrinol. (2000) 14:783–94. 10.1210/mend.14.6.0446 10847581

[79] Laplante M Sabatini DM. mTOR signaling in growth control and disease. Cell. (2012) 149:274–93. 10.1016/j.cell.2012.03.017 22500797 PMC3331679

[80] Ibrahim J Nguyen AH Rehman A Ochi A Jamal M Graffeo CS. Dendritic cell populations with different concentrations of lipid regulate tolerance and immunity in mouse and human liver. Gastroenterology. (2012) 143:1061–72. 10.1053/j.gastro.2012.06.003 22705178 PMC3459067

[81] Choi J Ravipati A Nimmagadda V Schubert M Castellani RJ Graffeo CS. Potential roles of PINK1 for increased PGC-1alpha-mediated mitochondrial fatty acid oxidation and their associations with Alzheimer disease and diabetes. Mitochondrion. (2014) 18:41–8. 10.1016/j.mito.2014.09.005 25260493 PMC4911223

[82] Maroof A English NR Bedford PA Gabrilovich D Knight SC. Developing dendritic cells become lacy cells packed with fat and glycogen. Immunology. (2005) 115:473–83. 10.1111/j.1365-2567.2005.02181.x 16011516 PMC1782181

[83] Kim J Byun JW Choi I Kim B Jeong H Jou I. PINK1 deficiency enhances inflammatory cytokine release from acutely prepared brain slices. Exp Neurobiol. (2013) 22:38–44. 10.5607/en.2013.22.1.38 23585721 PMC3620457

[84] Sliter DA Martinez J Hao L Chen X Sun N Fischer T. Parkin and PINK1 mitigate STING-induced inflammation. Nature. (2018) 561:258–62. 10.1038/s41586-018-0448-9 30135585 PMC7362342

[85] Matheoud D Sugiura A Bellemare-Pelletier A Laplante A Rondeau C Chemali M Parkinsons disease-related proteins PINK1 and Parkin repress mitochondrial antigen presentation. Cell. (2016) 166:314–27. 10.1016/j.cell.2016.05.039 27345367

