# Supplementary material for: Dendritic Cells Require PINK1-Mediated Phosphorylation of BCKDE1α to Promote Fatty Acid Oxidation for Immune Function
Source: Front Immunol. 2019 Oct 15;10:2386. doi: 10.3389/fimmu.2019.02386 (PMC6803436; doi:10.3389/fimmu.2019.02386)
Supplement: Supplementary file 1 [file Table_1.pdf]

Supplementary Table. 1. List of primers used for qPCR

Gene	Forward primer	Reverse primer
PGC1α	5′-GTAATCTGCGGGATGATGG-3′	5′-AATTGCTTGCGTCCACAAA-3′
NDUFA10	5′-TGGCTCAAGCAGGACAATCGCA-3′	5′-AGACACGGTCAGTCTGATGAGC-3′
CPT1A	5′-TGAGCGACTGGTGGGAGGAG-3′	5′-GAGCCAGACCTTGAAGTAGCG-3′
HADHα	5′-GCTAGACCGAGGACAGCAAC-3′	5′-CCTGCTTGAGACCAACTGCT-3′
HSL	5′-TACCGCAGCCTAGTGCACAC-3′	5′-AGATGGTCTGCAGGAATGGC-3′
PINK1	5′-GGACGCTGTTCCTCGTTA-3′	5′-ATCTGCGATCACCAGCCA-3′
GLUT1	5′-CCTGCAGTTTGGCTACAACA-3′	5′-GTGGACCCATGTCTGGTTG-3′
β-ACTIN	5′-GCAAGAGAGGCATCCTCACC-3′	5′-CGTAGATGGGCACAGTGTGG-3′
