# Supplementary material for: Dendritic Cells Require PINK1-Mediated Phosphorylation of BCKDE1α to Promote Fatty Acid Oxidation for Immune Function
Source: Front Immunol. 2019 Oct 15;10:2386. doi: 10.3389/fimmu.2019.02386 (PMC6803436; doi:10.3389/fimmu.2019.02386)
Supplement: Supplementary file 2 [file Data_Sheet_1.docx]

**Supplementary Figure. 1.** (A) Data was collected within same experiments as Figure 1D, but is shown separately for better understanding. Data represents mean ± SEM of three independent experiments performed in triplcate. ***p < 0.001 (two-way ANOVA). (B) Flow cytometry histograms of DCs stimulated with pRNA for 12 and stained with 2-NBDG. One experiment of 3 is shown (C) Flow cytometry histograms of DCs stained with Fixable Viability Dye eFluor™ 780. DCs were stimulated with pRNA for 12 h in the presence or absence of either 10 nM rotenone or 10 nM antimycin A. One experiment of 3 is shown. (D) Data was collected within same experiments as Figure 3G, but is shown separately for better understanding. Data represents mean ± SEM of three independent experiments performed in triplicate. *p < 0.05; **p < 0.01; ***p < 0.001 (Student's *t*-test). (D) Data was collected within same experiments as Figure 4B, but is shown separately for better understanding. Data represents mean ± SD of three independent experiments performed in triplicate. **p < 0.01; ***p < 0.001 (Student's *t*-test). (E) Flow cytometry histograms of DCs stained with Fixable Viability Dye eFluor™ 780. DCs were stimulated with pRNA for 12 h in the presence or absence of either 50µM BT2 or 20µM kinetin. (G) Percentage mean flouresence intensity of maturation markers (CD40 and CD80) in Dexa-DC. Data represents mean ± SD of three independent experiments performed in triplicate. **p < 0.01 (Student's *t*-test).

**Supplementary Figure 2. PINK1 deficient DC stimulate glycolysis for ATP production and cell survival.** (A) Flow cytometry dot plots of DCs stained with Fixable Viability Dye eFluor™ 780. siControl and siPINK1 DCs were stimulated with pRNA for 12 h. (B) Intracellular ATP levels were measured in DCs either transfected with siControl or siPINK1 unstimulated or stimulated pRNA for 12 h. Data represents mean ± SD of three independent experiments performed in triplicate. **p < 0.01 (Student's *t*-test). (C) mRNA levels were analyzed after 12 h of pRNA stimulation by (qPCR) and normalized to β-actin expression by using the 2^ΔΔCT^ method. Data represents Mean±SD of three independent experiments performed in triplicate. ***p < 0.001 (Student's *t*-test). (D) Flow cytometry histograms and percentage mean fluorescence intensity of DCs stained with 2-NBDG. Data represents Mean±SD of three independent experiments performed in triplicate. **p < 0.01; ***p < 0.001 (two-way ANOVA). (E) Glycolysis stress test of DCs trasnfected with either siControl or siPINK1 were stimulated for 12 h. Data represents Mean±SD of three independent experiments. (F) Data was collected within same experiments as Supplementary Fig. 2E, but is shown separately for better understanding. Data represents mean ± SD of three independent experiments performed in triplicate. **p < 0.01; ***p < 0.001 (two-way ANOVA). (G) APT production rates were calculated from mitochondrial fitness test (Fig. 2G) and glycolysis stress test (Supplementary Fig. 2E) were calculated and are expressed are percentage of relative change from unstimulated siControl DCs. Data represents Mean±SD of three independent experiments performed in triplicate. **p < 0.01; ***p < 0.001 (Student's *t*-test).

**Supplementary Information: Survival of PINK1 knockdown dendritic cells is dependent on glucose metabolism instead of OXPHOS**

Previously, PINK1 is described to be crucial for survival (Petit et al. 2005; Klinkenberg et al. 2010). However, the viability of PINK1 knockdown DC was not affected neither in the presence nor absence of pRNA (Supplementary Fig.2A). Furthermore, it is shown that PINK1 deficiency results in less ATP due to impaired mitochondrial fission leading to defective assembly of the ETC complexes, reduction in mitochondrial respiration and mitochondrial buffering capacity (Liu et al. 2011; Morais et al. 2009; Gegg et al. 2009; Amo et al. 2011). Total intracellular ATP levels were increased in both pRNA maturation of DC and PINK1-knockdown DC (Supplementary Fig.2B). We hypothesize that PINK1 knockdown DC maintain cell viability by reprogramming bioenergetics from OXPHOS to glycolysis.

To prove that PINK1 knockdown DC switch to glycolysis, we determined the expression of glucose transporter (Glut) *Glut1*, previously described to be of important for glycolysis in immune cells (Piatkiewicz et al. 2016; Freemerman et al. 2014; Macintyre et al. 2014). *Glut1* did not change upon pRNA maturation of DC. PINK1 knockdown cells significantly upregulated *Glut1* underscoring the switch to glycolysis (Supplementary Fig.2C). Glucose uptake, measured by 2-NBDG uptake, was not affected by pRNA maturation of DC (Supplementary Fig.2D). PINK1 knockdown significantly increased 2-NBDG uptake in DC (Supplementary Fig.2D) and this was significantly enhanced by pRNA maturation (Supplementary Fig.2D), indicating increased glycolysis in PINK1 knockdown DC. To determine the glycolytic activity, extracellular acidification rate (ECAR) was determined (glycolysis stress test). pRNA stimulation did not increase glycolysis and glycolytic capacity in DCs (Supplementary Fig. 2E-F). In contrast, PINK1 knockdown significantly increased glycolysis and glycolytic capacity in DC, and this was even enhanced by pRNA maturation (Supplementary Fig. 2E-F). The relative contribution of OXPHOS and glycolysis to ATP production in DC is calculated from the glycolysis stress test (Supplementary Fig. 2E) and mitochondrial stress test (Fig.2G). OXPHOS contributed to enhanced ATP production in pRNA-matured DC (Supplementary Fig. 2G). Conversely, glycolysis provided ATP in PINK1 knockdown DC in the presence and absence of pRNA (Supplementary Fig. 2G). Collectively, this shows that OXPHOS provides ATP upon pRNA maturation and loss of PINK1 promotes glycolysis in DC to maintain cell viability.
