# Supplementary figures and images for: Dendritic Cells Require PINK1-Mediated Phosphorylation of BCKDE1α to Promote Fatty Acid Oxidation for Immune Function
Source: Front Immunol. 2019 Oct 15;10:2386. doi: 10.3389/fimmu.2019.02386 (PMC6803436; doi:10.3389/fimmu.2019.02386)

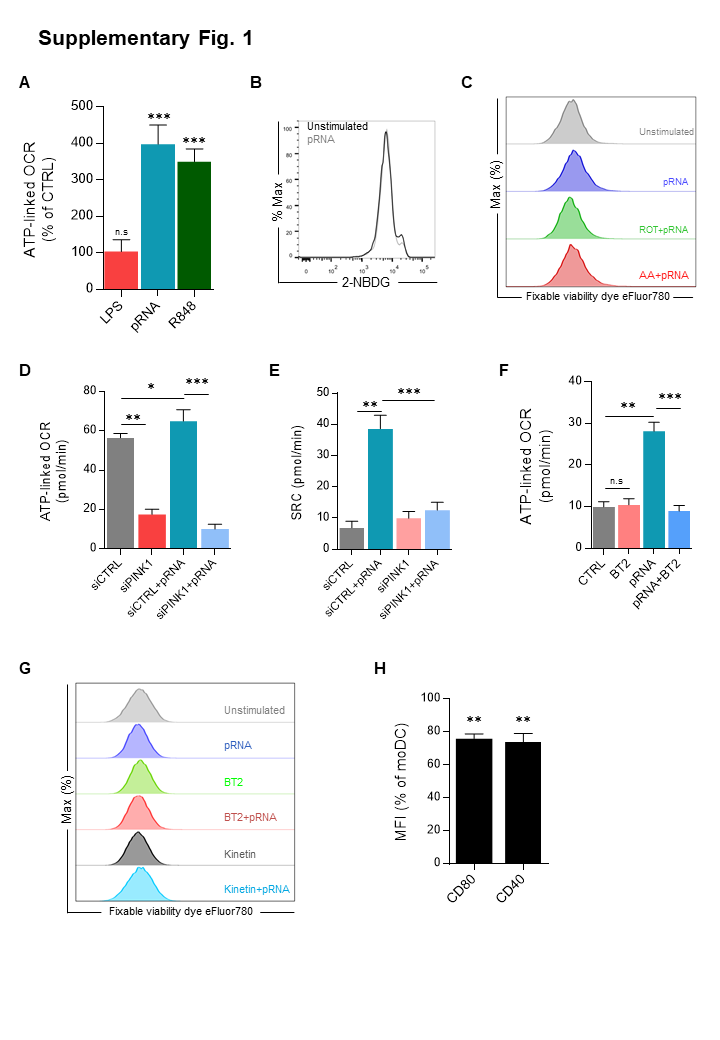

Supplement: Supplementary file 3 [file Image_1.tif]

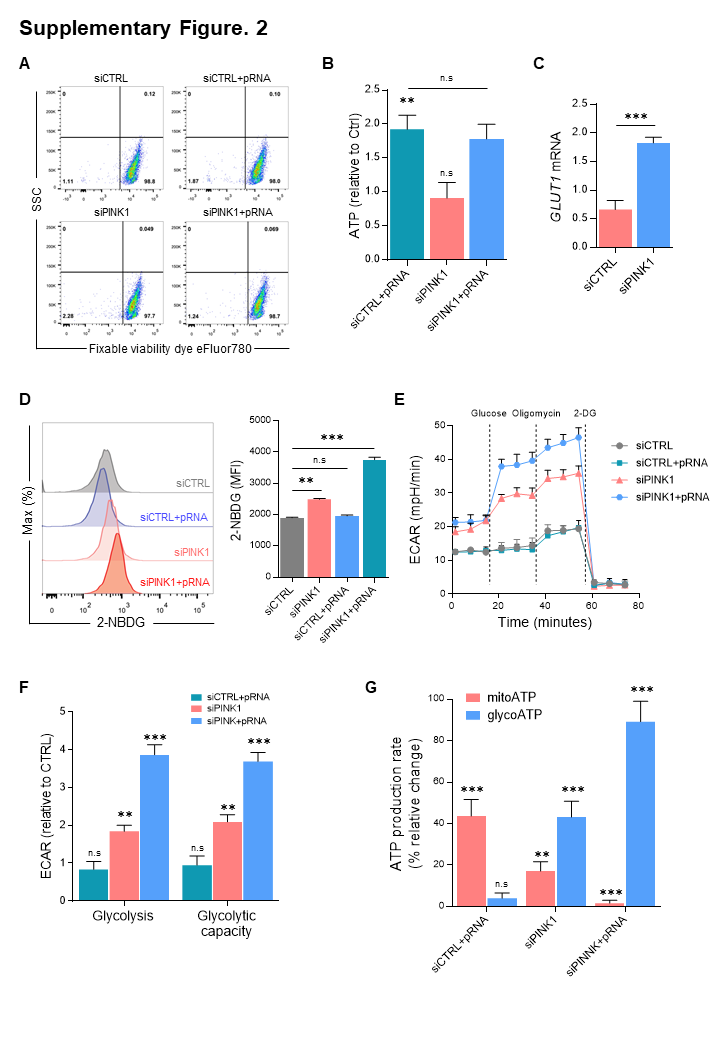

Supplement: Supplementary file 4 [file Image_2.tif]
